# Testing Persistence of Cohort Effects in the Epidemiology of Suicide: an Age-Period-Cohort Hysteresis Model

**DOI:** 10.1371/journal.pone.0158538

**Published:** 2016-07-21

**Authors:** Louis Chauvel, Anja K. Leist, Valentina Ponomarenko

**Affiliations:** Institute for Research on Socio-Economic Inequality (IRSEI), University of Luxembourg, Esch-sur-Alzette, Luxembourg; University of Vienna, School of Psychology, AUSTRIA

## Abstract

Birth cohort effects in suicide rates are well established, but to date there is no methodological approach or framework to test the temporal stability of these effects. We use the APC-Detrended (APCD) model to robustly estimate intensity of cohort effects identifying non-linear trends (or ‘detrended’ fluctuations) in suicide rates. The new APC-Hysteresis (APCH) model tests temporal stability of cohort effects. Analysing suicide rates in 25 WHO countries (periods 1970–74 to 2005–09; ages 20–24 to 70–79) with the APCD method, we find that country-specific birth cohort membership plays an important role in suicide rates. Among 25 countries, we detect 12 nations that show deep contrasts among cohort-specific suicide rates including Italy, Australia and the United States. The APCH method shows that cohort fluctuations are not stable across the life course but decline in Spain, France and Australia, whereas they remain stable in Italy, the United Kingdom and the Netherlands. We discuss the Spanish case with elevated suicide mortality of cohorts born 1965–1975 which declines with age, and the opposite case of the United States, where the identified cohort effects of those born around 1960 increase smoothly, but statistically significant across the life course.

## Introduction

A growing body of research investigates birth cohort effects in suicide rates [1–6]. While there is large international evidence that certain cohorts experience a higher risk of suicide compared to other birth cohorts, there is no knowledge if this higher risk is stable across the cohorts’ life course or if the risk that elevated suicide rates are capturing is only temporary. Further, no method is able to resolve the linear age-period-cohort relation. Even modern statistical models [7, 8] suffer from intrinsic problems to disentangle linear age, period, cohort (APC) effects [9–12]. In order to estimate the contribution of birth cohort effects apart from period and age effects in suicide rates, it is thus necessary to investigate the non-linearities of cohort effects (colloquially speaking the ‘cohort bumps’) as stable long–term deviations from a linear trend of social change, similar to the ‘zero linear trend’ (ZLT) [9]. We will investigate these non-linearities of cohort effects with a recently introduced APC-Detrended (APCD) approach [13]. APCD is useful to detect, test and internationally compare cohort contrasts where more gifted birth cohorts are systematically above the inter age-period-cohort trend compared with birth cohorts below the trend (see further elaboration in the methodological section). Further, with our advancement of APCD, APC-Hysteresis (APCH) that will be introduced here, we will estimate if these non-linear cohort effects are temporally stable or follow differential trajectories.

The need to disentangle age, period and cohort effects on social, economic and health outcomes has led to greater focus on age-period-cohort models. Cohort membership matters indeed in explaining different social phenomena, as shown for mortality rates [14], political behaviour [15], income position [13], educational attainment [16], suicide [17, 18] and other health outcomes [19]. Increases in cohort mortality by suicide have been shown for cohorts born after World War II in Britain [1], the United States [2] and Spain [3]. Similar results were found for pre-war cohorts in Australia [4], Brazil [5] and Quebec [6]. However, these models lack ability to control for confounders or draw causal conclusions.

Methodological developments to overcome these issues included, for instance, multi-country comparisons [20] and mixed models with random cohort effects that confirmed cohort effects in suicide and homicide rates [21], and more specifically, that youth suicide rates of larger cohorts and those with higher non-marital birth rates are larger [22, 23]. However, those approaches were not able to distinguish whether observed cohort characteristics indeed produced the cohort effect or simply mirror an unobserved cohort characteristic. By applying age-period-cohort models to overcome limitations of age-period models, cohort effects for suicide have been investigated in several studies [24, 25], but could not test the stability of these effects.

Well-debated in the scientific community, for instance with regard to the APC-Intrinsic estimator (APC-IE) [8], is the inherent difficulty to extract linear patterns of age, period and cohort effects [9–11]. Other problems involve choice of model constraints, hierarchies or substitutions to solve the identification problem that may seem arbitrary or convenience-driven [10, 11, 13]. If the identification of age, period, cohort non-linear fluctuations are methodologically feasible [26], linear trends generally do not contain meaningful information. Consequently, the recently established APCD method [13] focuses on non-linear fluctuations, i.e., ‘detrended’ cohort effects above and below the linear trend. It enables not only the inclusion of independent variables, but also post-estimation statistics. To sum up, the first aim of the present study is to robustly estimate non-linear cohort effects determining *intensity* of cohort contrasts, as APCD provides stable validated results independent of subjective choice of constraints. Here, after providing large-scale comparative multi-country evidence on variations in suicide rates, the second aim is to analyse the temporal *stability* of the non-linear effects. Therefore, we differentiate four potential trajectories.

In the sociological tradition of investigating suicide not as individual behaviour, but rather via collective experience and behaviour, we study the development of suicide risk by cohorts over their life course. Durkheim hypothesised *anomic suicide* as result of lack of social integration and social regulation [27]. Merton referred to *antisocial behaviour* due to relative frustration of the perceived divergence of aspirations and reality [28]. From this perspective, a collective social trauma due to exposure to unemployment, economic downturn, or poverty may lead to long-term increases in suicide risk. However, those assumptions in APC analyses are hardly testable. What we can test though is the stability of these effects.

Most prominent among possible trajectories is *stability* or ‘*hysteresis*’, well-illustrated by the scarring effect of early unemployment generating negative long-term outcomes to its victims [29, 30]. Indeed, Clark and colleagues used the term hysteresis to describe the process of long-term habituation to an initially negative condition, even if that condition may change or even vanish with time. Hysteresis can in the same sense be used to describe cohort processes of habituating to initially negative conditions, i.e. holding behaviour constant over time, even under changing period influences.

Persistence of cohort-specific risks, such as unemployment and lower chances of future employment of affected cohorts, known as *scarring effect* [29, 31], has up to now rather been hypothesised than clearly demonstrated and we test stability of scarring effects against other plausible trajectories of cohort effects. The second suggested trajectory is that of increasing cohort contrasts across the life course drawing from the theory of ‘*cumulative disadvantage*’ [32], assuming that earlier disadvantage prevents future access to certain resources or positions leading to an even greater deprivation in future life, the so-called ‘Matthew effect’ [33]. This increase in contrasting cohort trends results in ever increasing inequalities across the life course. The third trajectory is ‘*compensation*’, where initially high levels of suicide are compensated and diminish over the life course [34]. Fourth, early disadvantage could theoretically even lead to later advantage over other birth cohorts in the sense of a complete reversal or ‘*inversion*’ [35]. Bell [36] presented evidence of this pattern of inversion for subjective well-being in a British sample. In this paper, we intend to investigate these four differential trajectories by analysing (extreme) health risks of birth cohorts.

## Materials and Methods

### Data

We use data on suicide mortality from the WHO mortality database (http://www.who.int/healthinfo/mortality_data/en) for years 1970 to 2009 and for 25 countries. No ethical consent for the study was necessary since the data were analysed anonymously (mortality by cause data). From the WHO data sets, cause of death, country, gender, and population size of the country were retained for the male population. All considered data of the mortality database code causes of mortality with ICD 8 to ICD 10 (although classifications of causes of death were restructured through the ICD versions, classification of suicide was consistent through the versions). With age at death i and year of death j, we construct birth cohort k. We build eight five-year periods and five-year age groups from 20–24 to 75–79. We provide the Stata do file (S1 Dofile), dataset (S1 Dataset), and codebook (S1 Codebook) to replicate the analyses presented here.

### Strategy of data analysis

Relying on the tradition of age-period-cohort models, a dependent variable is explained by combination of age *a* (variable α_a_), cohort membership *c* (variable γ_c_) and period of measurement *p* (variable π_p_). This leads to the equation:

$${\text{y}}^{\text{apc}}=\;\mu \;+{\;\alpha }_{\text{a}}+{\;\pi }_{\text{p}}+{\;\gamma }_{\text{c}}\;\left(\text{APC}\right)$$

Before elaborating on APC models in more detail, three hypothetical configurations of suicide rates across time and cohorts illustrate the decomposition of age, period, cohort effects. Table 1 shows a data structure with *linear trends* which are impossible to attribute to period or cohort. To this data structure in Table 2 a *non-linear cohort effect* is added, which can be detected by APCD. Finally, data structure of Table 3 reflects *temporal change in the cohort effect*, showing a cohort effect at the beginning of the observed time period which vanishes across the life course, detectable by APCH. We first elaborate on linear trends shown in Table 1. While many papers proposed empirical estimations of the three effects [26, 37], an “identification problem” besets all APC models [38, 39]: an infinite number of solutions can fit the same outcome with equal efficiency, resulting from the relation *a* = *p—c*. That is, each variable is a combination of the other two. While in Table 1 the increase in suicide rates is obvious, the different possible effects cannot be disentangled, as the increases can either be understood as combination of age×period (age effect: suicide rate increases by 5 units per 5-year age group; period effect: suicide increases by 1 unit per 5-year period) or combination of age×cohort (age effect: again an increase of 5 units per 5-year age group; cohort effect: 1 unit more for each cohort born five years later), or one of the infinite more complex APC combinations. Despite some attempts to identify these linear trends [19, 40], no statistical model can overcome this intrinsic indetermination [10, 41, 42].

**Table 1:** Hypothetical data structure of suicide rates across time and cohorts: APC linear cohort effect. *Note*. a/y—age groups versus period.

a\y	1985	1990	1995	2000	2005
25	5	6	7	8	9
30	10	11	12	13	14
35	15	16	17	18	19
40	20	21	22	23	24
45	25	26	27	28	29
50	30	31	32	33	34
55	35	36	37	38	39

**Table 2:** Hypothetical data structure of suicide rates across time and cohorts: Non-linear cohort effect with persistent intensity. *Note*. a/y—age groups versus period.

a\y	1985	1990	1995	2000	2005
25	4.4	10.9	6.6	3	10.3
30	5.1	10.4	16.9	12.6	9
35	16.5	11.1	16.4	22.9	18.6
40	24.5	22.5	17.1	22.4	28.9
45	22.5	30.5	28.5	23.1	28.4
50	25.8	28.5	36.5	34.5	29.1
55	38.1	31.8	34.5	42.5	40.5

**Table 3:** Hypothetical data structure of suicide rates across time and cohorts: Temporal change in intensity of cohort effect. *Note*. a/y—age groups versus period.

a\y	1985	1990	1995	2000	2005
25	4.4	10.9	6.6	3	10.3
30	5.9	10.5	16	12.6	9.8
35	16	12.7	16.6	21.2	18.7
40	22.2	21.7	19.5	22.7	26.4
45	24.1	27.5	27.5	26.3	28.8
50	29.3	30.5	32.7	33.2	33.1
55	35	36	37	38	39

The second table (Table 2) superimposes a non-linear sinusoidal birth-cohort transformation. Those non-linear fluctuations had been constructed as waves that strictly follow a birth-cohort structure: some cohorts face higher risks at any age (e.g., cohorts born in 1945 and in 1965). APCD is able to extract cohort effect coefficients representing fluctuations above and below linear combination of age and period effects.

Table 3 illustrates hypothetical temporal change in the cohort effect by combining non-linear deviations in young cohorts (Table 2) and a linear trend in more senior age (Table 1), meaning that an initially observed cohort effect vanishes across the life course. In many empirical cases, e.g., for unemployment, unstable non-linearities can be observed when fluctuations are strong for young and weak for seniors. Initial cohort effects are thus completely absorbed over the analyzed lifespan. APCH is able to extract cohort fluctuations like APCD but extends the analysis by assessing the temporal stability or instability of the cohort effect with, but it also measures, with a hysteresis coefficient *h* (see section APCH and ‘hysteresis’ coefficient).

The methodology improvements here include three steps. The first is a Poisson specification of APCD originally built for ordinary least squares (OLS) [13] or logit specifications [43]. Second, a ‘cohortality coefficient’ is developed to express the degree to which birth cohort is a relevant expression of age×period. Third, APCH is built to assess cohort effect persistence.

### APCD in Poisson specification

First, a former OLS-type APCD model [13] is transformed into Poisson specification. For each country, a dependent variable *r*^*apc*^ denotes suicide rate (ratio between counts of suicides *d*^*apc*^ in reference population of size *n*^*apc*^) in age group *a* of period *p* belonging to cohort *c* = *p* − *a*. Categorical time effect variables pertaining to age effects *α*_*a*_, period effects *π*_*p*_ and cohort effects *γ*_*c*_, are then indexed by age *a*, period *p* and cohort *c*. In more detail, *a*, *p* and *c* are entangled in a two-dimensional space so two trend variables (rescale *a* and *c*) are added to absorb these linear trends. Three vectors representing age, period, cohort are constrained to sum = zero and slope = zero to reflect non-linearities. The first (*k* = 1) and the last (*k* = *a* + *p—*1) cohorts are omitted as they appear just once and their introduction substantially increases standard errors. The resulting model with only one solution provides fits relatively close to those of the APC-Intrinsic Estimators of Yang et al. [8] in many different contexts, except that the cohort effect in APCD shows a slope zero by construction. APCD is expressed in the following:

(APCD)

$$\{\begin{array}{c} log\left(r_{}^{apc}\right)=\alpha_{a}+\pi_{p}+\gamma_{c}+\alpha_{0}rescale\left(a\right)+\gamma_{0}rescale\left(c\right)+\beta_{0}+\varepsilon_{apc}\\ \{\begin{array}{c} \sum_{a}\alpha_{a}=\sum_{p}\pi_{p}=\sum_{c}\gamma_{c}=0\\ slope_{a}(\alpha_{a})=slope_{p}\left(\pi_{p}\right)=slope_{c}\left(\gamma_{c}\right)=0\\ with\;p=c+a\;and\;restricted\;to\;c_{min}<c<c_{max,} \end{array} \end{array}$$ (1)

where *α*_*a*_ is the age effect vector indexed by age group a, *π*_*p*_ is the period vector and *γ*_*c*_ is the cohort vector, *β*_*0*_ the constant. To provide a correct identification [14, 44], simple constraints such as Σ_*a*_*α*_*a*_ = Σ_*p*_*π*_*p*_ = Σ_*c*_*γ*_*c*_ = 0 imply centred coefficients. We add three constraints, *slope*_*a*_(*α*_*a*_) = *slope*_*p*_(*π*_*p*_) = *slope*_*c*_(*γ*_*c*_) = 0, where Slope is the linear function that gives the linear slope of the coefficients, so that *α*_*a*_, *π*_*p*_ and *γ*_*c*_ are detrended. The terms *α*_0_ Rescale(a) and *γ*_0_ Rescale(c) absorb the two linear trends of APC. Rescale is a transformation that standardizes the coefficients *α*_0_ and *γ*_0_: it transforms age from initial *a*_*min*_ to *a*_*max*_ to the interval −1 to +1. The constraint Slope_a_(α_a_) = 0 means the age coefficients *α*_*a*_, are trend-zero and is true only if Σ_a_ [(2*a* − *a*_*min*_ − *a*_*max*_)α_a_] = 0. This constraint is easily expressed as linear equation of coefficients. Coefficients *π*_*p*_ and *γ*_*c*_ are built analogously. This model is thus a traditional Mason and Smith [45] APC model, following Holford’s notion [41] that cohort is detrended as a constraint imposes zero slope on A, P and C effects.

If cohort effects do not exist, i.e. cohorts do not deviate from age and period characteristics, detrended cohort effect coefficients *γ*_*c*_ are zero: APCD provides no improvement compared with a simple age×period model (AP) with first and last cohorts omitted:

(AP)

$$\{\begin{array}{c} log\left(r_{}^{ap}\right)=\alpha_{a}+\pi_{p}+\alpha_{0}rescale\left(a\right)+\pi_{0}rescale\left(p\right)+\beta_{0}+\varepsilon_{ap}\\ \{\begin{array}{c} \sum_{a}\alpha_{a}=\sum_{p}\pi_{p}=0\\ slope_{a}(\alpha_{a})=slope_{p}\left(\pi_{p}\right)=0\\ \;restricted\;to\;c_{min}<c<c_{max.} \end{array} \end{array}.$$ (2)

However, if at least one *γ*_*c*_ coefficient is significantly different from zero (i.e., a significant non-linear effect deviating from the linear trend absorbed by AP), then AP is insufficient. Thus, to retain appropriate parsimonious models, comparing the Bayesian Information Criterion of (AP) and (APCD) is a diagnosis of relevance of non-linear cohort effects.

Under these specifications, APCD is a non-linear cohort effect detector of suicide, and *γ*_*c*_ is the detrended cohort effect (DCE) which assesses cohort non-linearities.

### ‘Cohortality’ coefficient

The *cohortality coefficient* (CC) assesses the degree to which the DCEs absorb (or do not) the full age×period interaction. CC is then a measure of relevance of cohort analysis. In an OLS specification, a natural index of appropriateness of cohort analysis consists of comparing (APC) and (AP), where age and period effects are summed. The full interaction (A×P) is also called “saturated model”, for example in sociology of mobility [46]. The gap between (AP) and (APC) is the addition of the diagonal interaction following birth cohorts; the one between (APC) and (A×P) is that the latter fits all cells of the interaction.

Then:

$$CC=\frac{R^{2}\left(APC\right)-R^{2}\left(AP\right)}{R^{2}\left(A\times P\right)-R^{2}\left(AP\right)}.,$$ (3.1)

where R^2^ is the coefficient of the determination of the model. Otherwise, CC is the percentage of residuals of the simple model (AP) explained by cohort effects. In a Poisson specification, the equivalent of R^2^ is the residual deviance statistics [47], so CC can be expressed as

$$CC=1-\frac{D\left(APC\right)}{D\left(AP\right)},$$ (3.2)

where:

$$\text{D}\left(model\right)\;=\;2\left(\text{ln}\left(L_{A\times P}\right)-\text{ln}\left(L_{model}\right)\right)\;\left(\text{L denotes the likelihood function}\right)$$ (3.3)

Again, in this Poisson compatible specification, CC measures how a cohort accurately summarizes the interaction between A and P. When CC is close to one, the cohort term perfectly fits the interaction. If CC is closer to zero, birth cohort is not relevant in explaining the phenomenon.

### APCH and ‘hysteresis’ coefficient

Next, the APCH model is built, which assesses persistence of cohort effects over the life span. APCH is designed to assess the degree of stability of cohort effects, and thus the ‘scarring’ of cohorts, by assessing the interaction between cohort fluctuations and age. Interested readers are referred to the original ado commands, as both APCD and APCH have been developed as STATA ado commands that can be downloaded with the usual ‘ssc install apcd’ and ‘ssc install apch’ orders. Because they are based on STATA glm, they can handle OLS, logit and Poisson log-linear specifications.

The principle of APCH is to test the degree of stability across the (observed) life course of detected significantly nonzero.*γ*_*c*_ The solution is to estimate *hysteresis coefficient h* to differentiate trajectories via APCH:

(APCH)

$$\{\begin{array}{c} log\left(r_{}^{apc}\right)\;=\alpha_{a}+\pi_{p}+\left(1+h.rescale\left(a\right)\right)\gamma_{c}+\alpha_{0}rescale\left(a\right)+\gamma_{0}rescale\left(c\right)+\beta_{0}+\varepsilon_{apc}\\ \{\begin{array}{c} \sum_{a}\alpha_{a}=\sum_{p}\pi_{p}=\sum_{c}\gamma_{c}=0\\ slope_{a}(\alpha_{a})=slope_{p}\left(\pi_{p}\right)=slope_{c}\left(\gamma_{c}\right)=0\\ with\;p=c+a\;and\;restricted\;to\;c_{min}<c<c_{max} \end{array} \end{array}$$ (4)

APCH is an advancement of APCD where the simple *γ*_*c*_ term is replaced by a variable term (1+*h*.*rescale*(*a*)) *γ*_*c*_. Negative values of *h* represent diminishing cohort fluctuations detected by *γ*_*c*_: the ‘cohort effect’ is changing across the life course. There, *γ*_*c*_ captures the middle-age value of the cohort effect, somehow an average cohort effect that culminates when rescale(a) is minimum (rescale(*a*) = −1 at the beginning of the observed life span), and the cohort effect diminishes (when *h* < 0) at the end of the observed life span (when rescale(*a*) = +1). More precisely, APCH offers calibration of *h* where, if *h* = −1, cohort effect *γ*_*c*_ is an average between its maximum at entry and a null effect at the end of the observed period. The detected cohort non-linearity can thus either relate to stable or instable trajectories, which correspond to the spectrum of possible *h* values as follows:

- *h* > 0 represents increasing cohort effects across the life course (*cumulative disadvantage*);
- *h* = 0 is cohort effect without specific trend and APCH is APCD (*hysteresis/stability*);
−1 < *h* < 0 describes compensation in that cohort fluctuations diminish over the life span; *h* can be understood as percentage measurement of the decline from rescale(*a*) = −1 at entry to rescale(*a*) = +1 at the end of observed lifespan (*compensation*);
- *h* ≤ −1 is *cohort inversion*.

Because of the non-linear type of interaction involved here, estimation of *γ*_*c*_ (the DCE coefficients) and of the *h* coefficient cannot be simultaneous. The strategy here is to build an iterative process of estimation where at step (*n*), we alternate a first substep (*n*,1) with estimation of the *h* of step (*n*) as an interaction between rescaled age and DCE of step (*n*−1) that had been previously estimated. At second substep (*n*,2) where the DCE of step (*n*) is now estimated based on the estimate of *h* of substep (*n*,1) and the [*h*.*rescale*(*a*) *γ*_*c*_] term estimated at step (*n*−1). At step (1), *h*(1) is estimated based on the results DCE(0) of the initial APCD.

APCH (*n*,1) will estimate the *hysteresis coefficient* ${\hat{h}}^{\left(n\right)}$.

$$\{\begin{array}{c} log\left(r_{}^{apc}\right)\;=\alpha_{a}+\pi_{p}+\left(1+h.rescale\left(a\right)\right){\hat{\gamma }}_{c}^{\left(n-1\right)}+\alpha_{0}rescale\left(a\right)+\gamma_{0}rescale\left(c\right)+\beta_{0}+\varepsilon_{apc}\\ \{\begin{array}{c} \underset{a}{\sum }\alpha_{a}=\underset{p}{\sum }\pi_{p}=0\\ slope_{a}(\alpha_{a})=slope_{p}\left(\pi_{p}\right)=slope_{c}\left(\gamma_{c}\right)=0\\ with\;p=c+a\;and\;restricted\;to\;c_{min}<c<c_{max.} \end{array} \end{array}$$ (5)

APCH (*n*,2) will estimate the DCE(*n*) ${\hat{\gamma }}_{c}^{\left(n\right)}$

$$\{\begin{array}{c} log\left(r_{}^{apc}\right)\;=\alpha_{a}+\pi_{p}+\gamma_{c}+\left({\hat{h}}^{\left(n\right)}.rescale\left(a\right)\right){\hat{\gamma }}_{c}^{\left(n-1\right)}+\alpha_{0}rescale\left(a\right)+\gamma_{0}rescale\left(c\right)+\beta_{0}+\varepsilon_{apc}\\ \{\begin{array}{c} \underset{a}{\sum }\alpha_{a}=\underset{p}{\sum }\pi_{p}=\underset{c}{\sum }\gamma_{c}=0\\ slope_{a}(\alpha_{a})=slope_{p}\left(\pi_{p}\right)=slope_{c}\left(\gamma_{c}\right)=0\\ with\;p=c+a\;and\;restricted\;to\;c_{min}<c<c_{max.} \end{array} \end{array}$$ (6)

This iterative process of estimation converges rapidly towards a unique result, i.e., a DCE and a *hysteresis* coefficient *h*. Testing the models with hypothetical data structures of Tables 1 to 3 provide respective coefficients in Table 4.

**Table 4:** Goodness-of-fit and relevance of cohort analysis for hypothetical data structures in Tables 1 to 3.

	APC linear cohort effect (Table 1)	Non-linear cohort effect with persistent intensity (Table 2)	Temporal change in intensity of cohort effect (Table 3)
CC	0.000	1.000	5
BIC AP	-115.0	308.8	57.9
BIC APC	-52.0	-52.4	-39.7
BIC APCH	-52.3	-52.0	-52.3
h	-	0.00	-0.97
h-st.err.	-	0.00	0.01

*Note*. CC—Cohortality coefficient; BIC—Bayesian Information Criterion; AP—age-period model; APC—age-period-cohort model; APCH—age-period-cohort hysteresis model; h—hysteresis coefficient; h st.err.–standard error of the hysteresis coefficient. Please note that smaller BICs indicate better model fit; for negative BICs, the more negative the better the model fit [48].

Resuming the data structures of Tables 1 to 3, Table 1 showed *linear trends* in suicide rates impossible to decompose, Table 2 an additional *non-linear cohort effect* detectable by APCD, and Table 3 *temporal change in the cohort effect*, i.e., a cohort effect at the beginning of the observed time period which vanishes across time, detectable by APCH. As depicted in Table 4, for *linear trends* (Table 1), CC = zero reflects irrelevance of cohort analysis; comparison of BICs between AP and APC show APC fits better. APCH does not improve APC. As DCE are not relevant in this example, no *h* coefficient is provided. For *persistent intensity of cohort effect* (Table 2), CC = 1 and the cohort fluctuation perfectly represents the *a*×*p* interaction. Comparing BIC_AP_ to BIC_APC_ shows that cohort is relevant but APCH is not further improving fit of the model, i.e. temporal change of the trend is no issue here: The value of *h* is not significantly different to zero: the DCE is stable across the life span. For *temporal change in intensity of cohort effect* (Table 3), CC = 0.9 reflects temporal change in cohort structure, confirmed by BIC comparison with best fit of APCH. The *h* coefficient is almost -1, expressing the non-linear cohort effect vanishing across the life course, reflecting a pattern close to the earlier, theoretically assumed *inversion* across life span. On other sets of simulations (http://www.louischauvel.org/apchex), these methodological choices return appropriate values of *h* and DCE. Standard error of *h* and BIC differences of the models can assess if APCD is preferred over AP, and if APCH is preferred over APCD.

## Results

### Descriptive step

**Fig 1:**
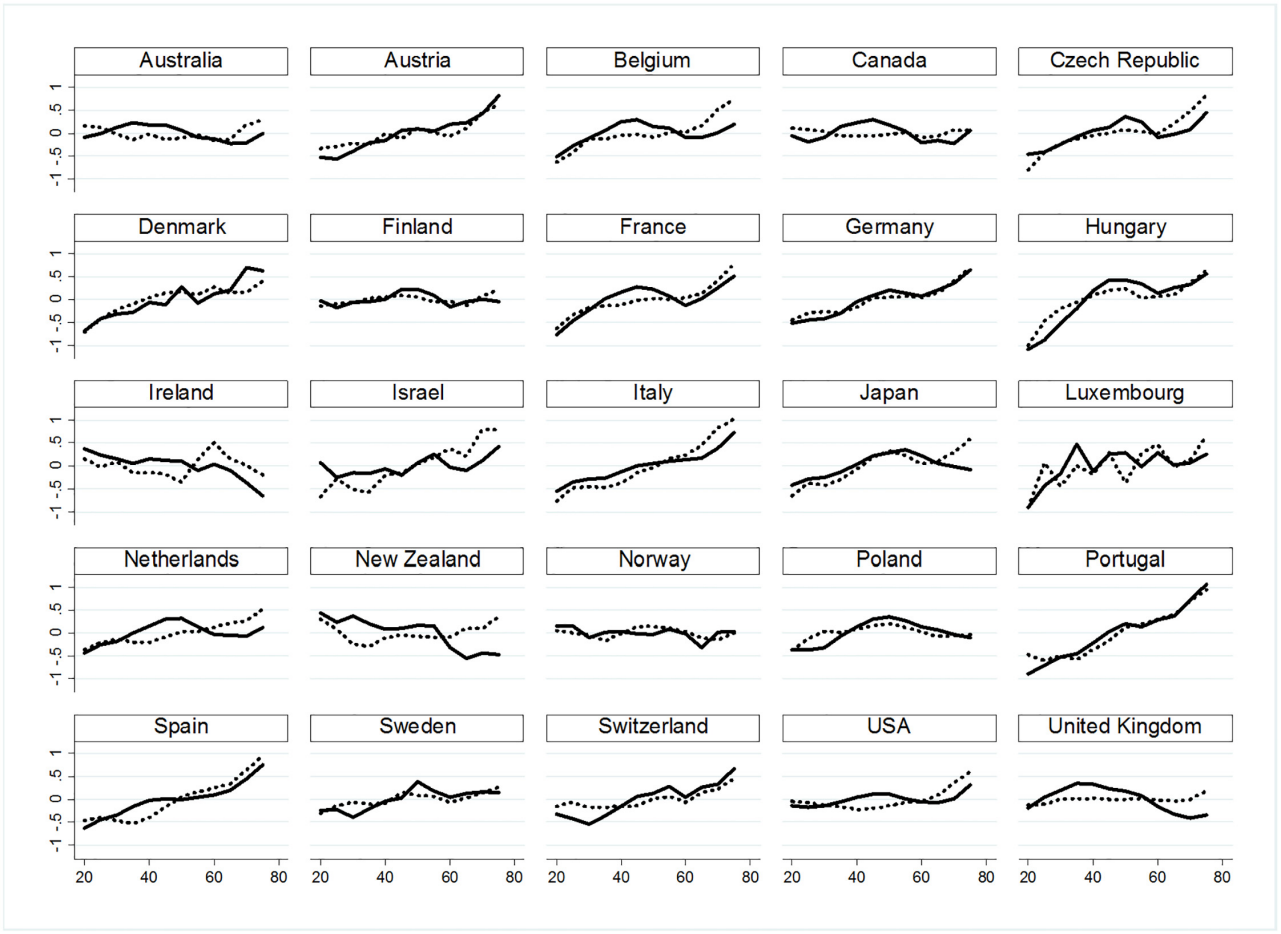
Transformation of the age profile of logged suicide rate in 1985 (dashed line) and 2005 (solid line).

A descriptive analysis of the interaction between age and period reveals relevant changes in the age profile of suicide rates. Whereas suicide for men in old age was typically very high, the age ratio is changing towards younger males. Fig 1 displays the changing age distribution of suicide in 1985 and 2005 for each country across periods. In 2005, compared with rates in 1985, suicide increased for younger ages in many countries. In Australia, Belgium, Canada, France, Italy, the Netherlands, Spain, the United States and United Kingdom, suicide in old age is less common in 2005 than in 1985. The opposite holds for youth and middle-aged populations where an age transformation in suicide is observed, i.e., suicide rates vary for different ages.

**Fig 2:**
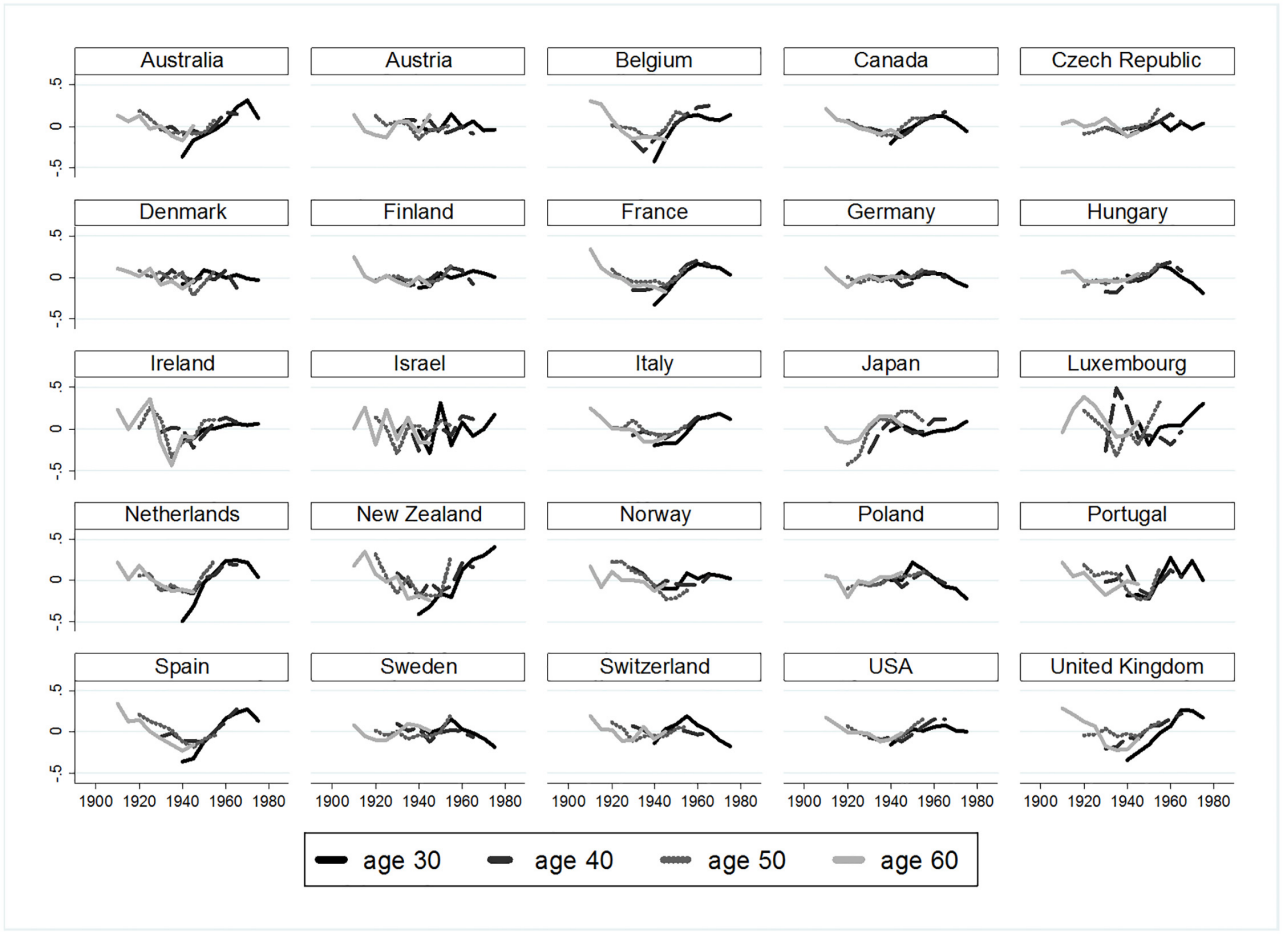
Residuals of logged suicide rates after control of (AP) by country.

**Fig 3:**
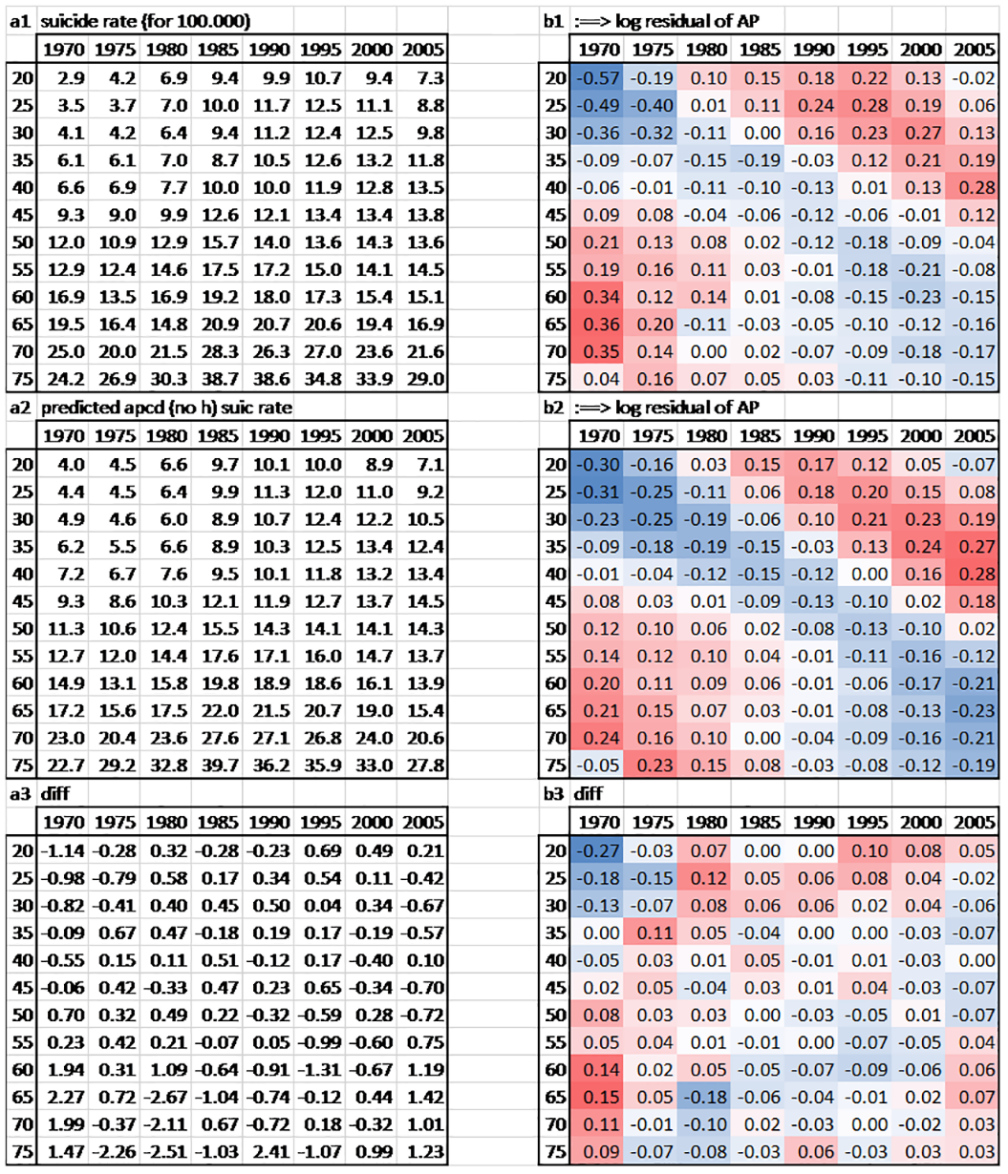
Hypothetical versus empirical suicide fluctuations: The Spanish case. *Note*. We compare three datasets: The actual empirical dataset *a1* (suicide rates per 100,000 population), a simulated dataset without changes in intensity of cohort effects (i.e. hysteresis/stability over the life course) *a2*, and the difference between cells *a3*. Now consider *b1* and *b2*, the residuals of the logged values of datasets *a1* and *a2* in the model (AP) (we suppress the linear combination of age and period dummies). *b1* shows some cohort effects, that are ‘imperfect’ compared to *b2* the expected values under the hypothesis of a pure APC without h effect. In these tables, the red cells are the high values of suicide and the blue cells the relatively protected groups. *b3* presents the differences between empirical *b1* and theoretical *b2*, and following the diagonally changing colours, we can detect temporal stability/changes of cohort fluctuations.

**Fig 4:**
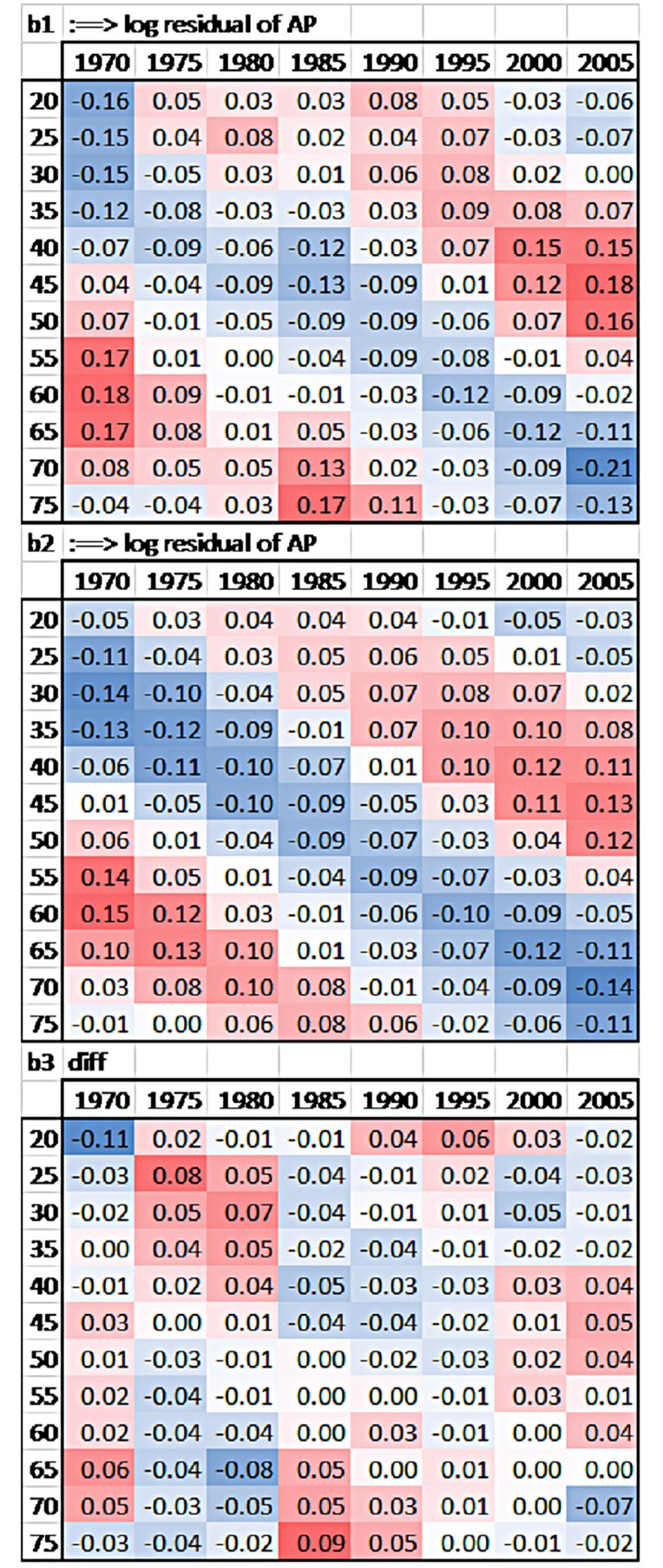
Hypothetical versus empirical suicide fluctuations: The U.S. case. *Note*. See legend of Fig 3 for explanations, for reasons of space only b1-b3 are presented.

To better understand interactions age×period in each country, residuals of the (AP) model show higher or lower suicide risks for each cohort (Fig 2), as opposed to the hypothesised null interaction of country-specific age×period (i.e., the age profile does not change over periods). Indeed, lines of the different age groups would be completely flat if no cohort effects existed, but we detect strong fluctuations which indicate cohort effects (Fig 2). For example, in Spain, old-age suicide decreased for cohorts born after 1920, but suicide at age 30 and 40 increased considerably for cohorts born after 1940 (see Fig 3 for an illustration with a hypothetical and the empirical dataset). The same development can be observed in Australia, Belgium, France, Ireland, New Zealand, the United Kingdom, and the United States (see Fig 4). These Figures simply reflect age and period descriptives, not a result based on age-period-cohort models. Fig 2 shows that specific cohorts are systematically above or below zero, showing specific cohort-based risks below and above the trend across the life course.

### APCD: Examining intensity of cohort effects

**Fig 5:**
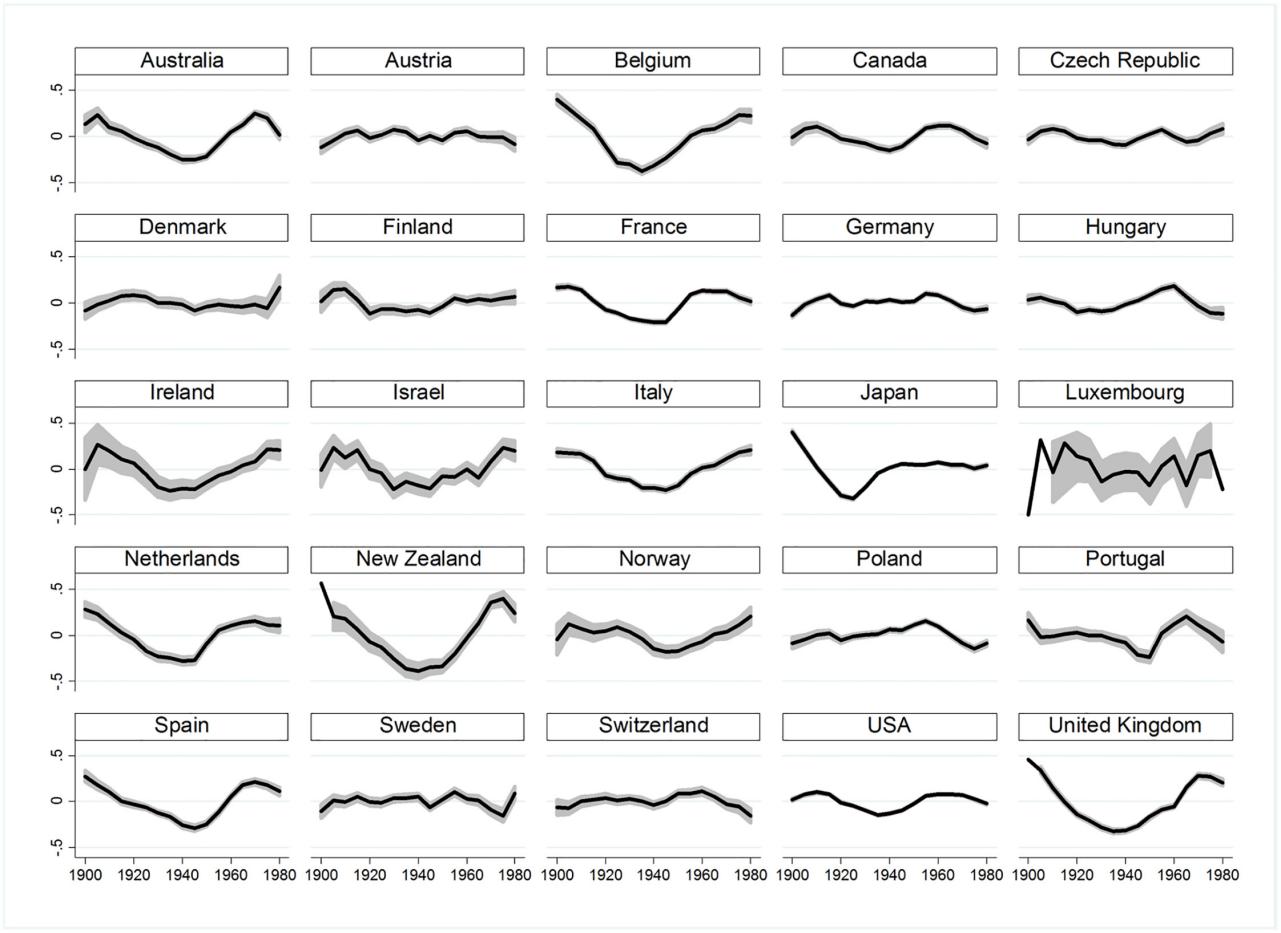
Cohort APCD coefficients: structure of cohort ‘bumps’ in male suicide. *Note*. 95% confidence intervals in grey.

After detecting cohort differences, their shape and intensity are displayed with APCD as detrended APC coefficients in Fig 5 with 95% confidence intervals and zero mean. Horizontal lines indicate very small detrended cohort effects. Germany, Austria and countries in Northern and Eastern Europe are characterized by less intensive non-linear cohort trends. For instance, for Austria, the age profile does not change and thus the DCEs do not show a cohort bump. Conversely, several countries show similar configurations with strong downward oscillations for cohorts born in 1940 to 1950: strong cohort fluctuations are found for Australia, Belgium, France, Italy, the Netherlands, New Zealand, Spain and the United Kingdom. In these countries cohorts born around 1940 are ‘luckier’ cohorts that are less likely to commit suicide when age, period and overall cohort linear trend effects are controlled for. By directly comparing age- and period-dependent patterns in suicide rates of different countries, particularly important fluctuations are detected in Belgium, New Zealand and the United Kingdom. They show massive variations with +0.4 to −0.4 standard deviations in Belgium for cohorts born between 1900 and 1950. This means that relative suicide rates vary by a magnitude of 1 to 2 among these cohorts at the same age. For countries with flat horizontal lines cohort analysis is not relevant as most of variation in suicide rates is due to age and period effects.

To conclude, strong cohort contrasts exist in Australia, Belgium, France, Italy, the Netherlands, New Zealand, Spain and the United Kingdom. In order to examine stability of these cohort effects, cohort difference intensity is measured by the standard deviation of the DCE coefficients, thus assessing ‘cohort contrasts’ in suicide rates and thus *intensity* of disadvantage or advantage of one birth cohort compared to others (that we will cross with the CC).

### Cohortality coefficient (CC): Identifying countries with consistent cohort effects

**Fig 6:**
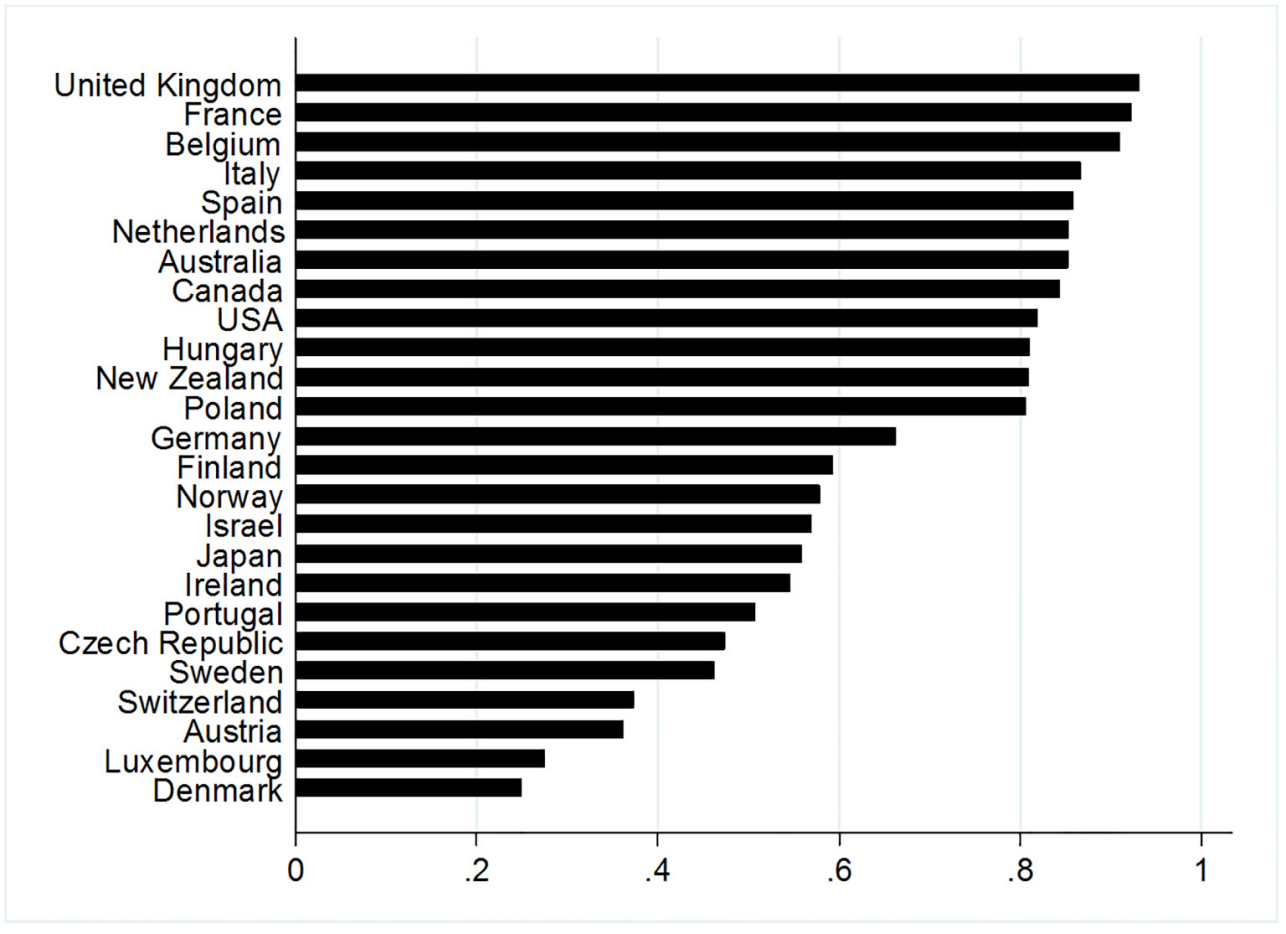
Cohortality coefficients of the 25 countries. The 12 countries with higher CC are retained for further analysis of hysteresis.

After identifying shape and significance of cohort effects (Fig 5), the CC tool detects cases where cohort is a consistent explanation of suicide rate, i.e., the extent to which the age-period interaction is due to cohort effects (Fig 6). Although no explicit rule has been introduced for a cut-off of the CC coefficient, based on this sample of countries we choose 0.8 (see also the gap in Fig 6 between CCs per country). This cut-off helps to run analyses parsimoniously by pre-selecting only those constellations where cohort fluctuations are present and can be tested for temporal stability (compare with Fig 2). CC is above 0.8 for the 12 upper cases, with Poland at the lower end, scoring just above 0.8, and the United Kingdom has the strongest CC at 0.93. Countries with CC below 0.8 show either no cohort effect (Austria, Germany), more complicated interactions of age, period, cohort in the sense of cohort fluctuations that are influenced by simultaneously occurring period or age effects, with Japanese cohorts born in the 1940s starting with high suicide rates becoming better protected and converse patterns for those born in the 1920s (see Fig 2), or shaky variations due to smaller national populations (Israel, Luxembourg).

### APCH: Detecting temporal stability of cohort effects

**Fig 7:**
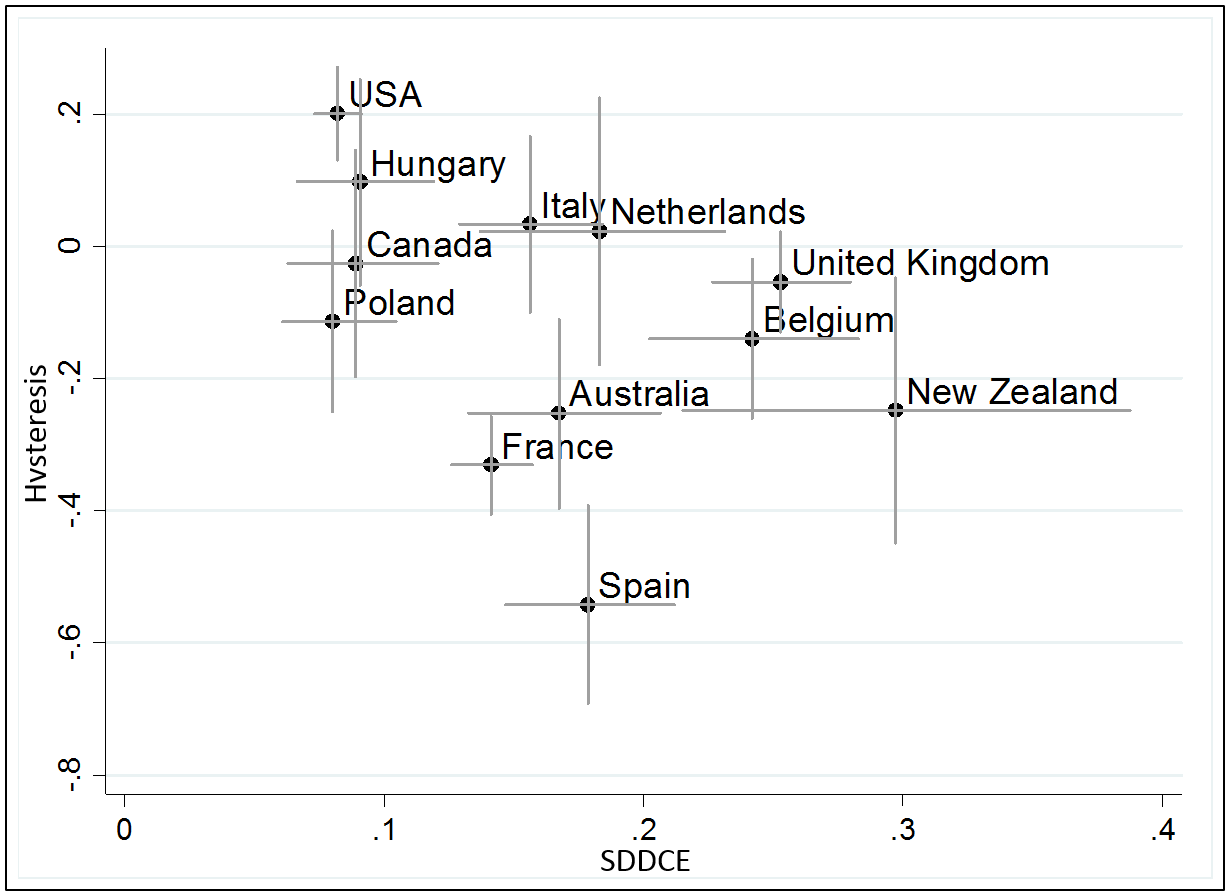
Standard deviation of the detrended cohort effects (SDDCE) and *h* coefficients in the 12 countries with higher Cohortality Coefficient.

The CC detects relevant cohort effects in 12 countries: Poland, New Zealand, Hungary, the United States, Canada, Australia, the Netherlands, Spain, Italy, Belgium, France, and the United Kingdom. However, high CC does not inform about the stability of the cohort effect. APCH detects degree of stability of cohort disadvantage and as such the development of cohort contrasts across time. Fig 7 show standard deviations of the DCEs plotted against hysteresis parameters of those countries with relevant cohort effects.

In Fig 7, standard deviation of the DCE coefficients and *h* plot shows the difference between countries with weak cohort effects (left) and stronger cohort effects (right). In case of the −1 < *h* < 0, the cohort effects decrease across the life course (lower side). In the case of *h* > 0, cohort effects increase, so the effect is cumulative (upper side). The spikes indicate the 95% confidence intervals on both variables. Below the baseline are countries where cohort effects decline across the life course questioning the implicit hypothesis of stability of cohort effects. Considering standard errors of estimates of *h*, the *hysteresis/stability* hypothesis is acceptable in only six of the 12 cases: Poland, Canada, Hungary, Italy, the Netherlands, and the United Kingdom.

Our analysis provides evidence for *cumulative disadvantage* in the United States as cohort fluctuations are increasing. On the contrary, the cohort effect of Spain can be interpreted as strong *cohort compensation*, and initially unlucky cohorts born 1965–1975 could partially recover with time. If *h* is significantly different from zero disadvantaged cohorts indeed remain disadvantaged however with only partial stability. For instance, in Belgium only 15% (*h* = -.15+/-.11) of disadvantage is absorbed across the life course. Additionally, one-quarter to one-third of cohort effects weaken in Australia, New Zealand and France. Therefore, rather than merely assuming persistence of cohort effects, the observed cases show the importance of considering the value of *h* to find evidence regarding actual stability of cohort contrasts.

Testing this APCH model against a random effects model as suggested by one reviewer, we arrive at similar results for a simulated data set (see syntax at http://www.louischauvel.org/simu_hapc_apch.do). For the empirical dataset used here, APCH provides the further advantage of providing robust results without requiring manual fine-tuning, arriving at stable and ready-to-use estimations of hysteresis (stability) coefficients of cohort effects via an automatized iterative process. Random effects models may be useful when year and cohort measured variables are of interest [49]. Bayesian estimation may solve convergence issues for random effects models.

## Discussion

The present study introduced a technique to disentangle non-linear cohort effects from age and period effects and to test their persistence across the life course. The APCD and the newly developed APCH method focus on non-linear effects net of any trends and provide evidence that cohort effects in suicide rates are relevant for some of the observed countries of which several are stable across the life course. Stable non-linear cohort effects are found for the United Kingdom, Italy and the Netherlands, whereas France and Spain show cohort fluctuations with decreasing intensity across the life course. Cohort disadvantages are significantly stable in six of 12 countries, whereas in the United States, cohort contrasts are smoothly but significantly increasing across life span.

### Explanation of findings

In the first part of this paper, intensity of cohort disadvantages changing across the life course was assessed by the *h* coefficient to distinguish trajectories of initial cohort disadvantages across the life course. Despite commonly hypothesised stability of cohort disadvantage, our analyses showed that among suicide rates of 25 countries, only six cases showed stable cohort fluctuations (Poland, Italy, the Netherlands, Canada, United Kingdom and Hungary). We can identify other configurations which prove the usefulness of our method of testing stability of cohort effects: *cumulative disadvantage* (the United States) and *compensation* (Spain).

In order to explain the strong suicide fluctuations for cohorts born 1965–1975 in Spain, with elevated levels in suicide mortality in the 1980s but those effects diminishing over time, we need to consider the specific economic and social context of Spain in the 1980s. Increases in suicide mortality during this time have been long documented [3]. The overall loss of life expectancy in young adults during the 1980s in Spain, rather than increase as would be expected, has been mainly attributed as consequence of HIV/AIDS, drugs and violent deaths, particularly traffic accidents [50]. This has been explained with very particular social and economic developments at that time in Spain: Whereas political and structural crises led to breaking with old norms and values, at the same time unemployment and labour market reforms prohibited the young from realizing their aspirations, which presumably led them to adopting unhealthy lifestyle behaviours such as alcohol and drug use, and driving under the influence of alcohol [50]. Unemployment rate has indeed been linked to elevated suicide mortality for Galicia, Spain [51]. This socioeconomic context coupled with wide availability of drugs and repeated use of injection needles led to the HIV outbreak, resulting in strong increase in AIDS mortality. Deaths classified as suicide at that time could also be attributable to the diagnosis and bad prognosis of AIDS [52]. The total loss of life expectancy for men and women due to HIV/AIDS, traffic accidents and suicide between 1980 and 1990 has been estimated to be 0.8 and 0.2 years, respectively, in Catalonia [53]. The negative h coefficient found for Spain mirrors the efforts of public health management to absorb those shocks specific to these generations after 1990, and afterwards became a public health issue that was not specific to those generations anymore.

The case of the United States [2, 18] is a specific example but it echoes a cohort structure relatively common in other Western countries; U.S. cohort fluctuations are significant but relatively modest compared to eight other nations. When the linear trend is taken into account, the luckier cohorts were less likely to commit suicide, with the luckiest cohort born 1920–1950. Hence, the ‘luckiest’ cohort members being those born around World War II or immediately after. As one reviewer points out, the positive h coefficient for the U.S. could have also been a chance finding due to multiple testing. However, we have replicated the U.S. findings with another data source: Merging Centers for Disease Control and Prevention (CDC) Multiple Cause of Death files matched with Current Population Survey (CPS) data of 1990–2010, we find the exactly same increase in mid-age mortality by suicide of the 1960-born cohort, more specifically those low educated, white, and not married [54]. We have extended those calculations by running the APC-H method on those new data as well and, mirroring the presented findings, found again a positive h coefficient in the CDC-CPS merged file. Although we cannot make use of further explanatory variables, it is likely that cohorts born 1920 to 1950 experienced post-war economic boom and context of full employment specific of affluent societies [55] in their youth and middle age. The strong positive h coefficient of the U.S. can be explained with rising socioeconomic stressors across the life course of the affected cohorts: The problems the baby boomers have been facing for some time were strongly increasing in the first decade of the 21st century. In comparison, cohorts born around 1960 could have been affected by economic slowdown of the 1980s. Those cohorts born around 1960, more specifically the non-Hispanic white male members of these cohorts, have recently been identified as especially vulnerable to morbidity and mortality [56], a finding which has been validated with age-period-cohort methodology for suicide, especially for low-educated and un-married members of the cohort [54]. Referring to theory of relative frustration, cohorts born around 1960 experienced a different economic situation in middle age than they expected. Where cohorts born 1900 to 1920 might have experienced material deprivation and scarcity of resources, cohorts born 1950 to 1975 could have perceived relative deprivation in comparison with their parents’ situation at their age. Higher suicide rates could be an extreme outcome of this divergence.

### Limitations and suggestions for further research

The analyses here use the WHO mortality database with a sample of 25 countries for a period of almost 40 years (1970–2009). Data on further countries and longer time frames (for some countries since 1950, newer data up to 2013 at the moment of writing) are available in the database. Hence it would be desirable if further research extends the presented analyses and tests presence and temporal stability of cohort effects for a larger sample of countries and even longer time spans. One should also note that with longer time frames under investigations, cohort dynamics may appear different overall because extreme conditions in the newly considered time interval (before or after the initially considered interval) may change the long-term cohort trajectory in some cases. Furthermore, additional methodological elaborations with regard to the different interactions of age, period and cohort may be considered relevant depending on the conceptual framework.

In some of the investigated countries, birth cohorts showed initial disadvantage but partial or even full recovery of disadvantage across the life course (*compensation*), whereas others remained disadvantaged (*stability*). However, searching for reasons for differential disadvantage and differential trajectories was beyond the scope of the paper. In further studies, it would be desirable to investigate causes for temporary or stable disadvantage. Future research should include both micro-level information on education and other socioeconomic indicators to investigate how birth cohort characteristics may ‘shape their disadvantage’, see a review of papers related to social stratification and suicide [57]. As the present study only included men it is necessary to investigate possible gender effects and possible interactions of age, period, cohort effects with gender. One advancement would be to provide hysteresis coefficients for each cohort separately, when this paper only considered the hysteresis of the overall society under investigation. Another further development of our method could be to disentangle slowly evolving cohort fluctuations (e.g. New Zealand, Fig 2) versus abruptly occurring cohort fluctuations (e.g. French baby boomers). Rapidly significantly fluctuating cohort contrasts means a strong differentiation of close cohorts. Conversely, slow U-shaped cohort fluctuations might hide more complicated evolutions, and be less diagnostic in identifying disadvantaged cohorts. The test of temporal stability of those cohort fluctuations however stays the same.

## Conclusions

Investigating temporal stability of suicide fluctuations in a set of 25 countries and between 1970 and 2009, we detect 12 nations where cohort fluctuations are unstable, in particular in Spain elevated suicide mortality of cohorts born 1965–75 declines with age, and in the U.S. cohort effects of those born around 1960 and protected at earlier ages increase across the life course.

Further research should include more information on historical economic situation to investigate contextual causes for temporary or stable disadvantage: Now that we can differentiate persistent and temporary cohort specificities in suicide rates, we will be able to study better the links between socio-economic context of socialization and temporal change of cohort fluctuations in suicidal behaviour.

## Supporting Information

S1 Codebook Codebook for WHO suicide mortality data. (DOCX)

S1 Dataset Stata dataset with suicide rates of WHO mortality database 1970–2009 for 25 countries. (DTA)

S1 Dofile Stata do file to replicate APCD and APCH models and figures. (DO)
