# Supplementary material for: Testing Persistence of Cohort Effects in the Epidemiology of Suicide: an Age-Period-Cohort Hysteresis Model
Source: PLoS One. 2016 Jul 21;11(7):e0158538. doi: 10.1371/journal.pone.0158538 (PMC4956228; doi:10.1371/journal.pone.0158538)
Supplement: S1 Codebook — (DOCX) [file pone.0158538.s001.docx]

**Testing persistence of cohort effects in the epidemiology of suicide: an age-period-cohort hysteresis model**

Chauvel, Louis, Anja K. Leist and Valentina Ponomarenko

Codebook on WHO suicide data (ICD8 to ICD10)

We provide the WHO ICD8 to ICD10 data on suicide mortality. The WHO mortality data base makes annual data on causes of death by country, age and sex available and can be downloaded at http://www.who.int/healthinfo/mortality_data/en/ (last version of November 2013). We combined the data from ICD8 to ICD 10 of suicide mortality. The classification death due to “self-inflicted harm” is consistent throughout the versions. Hence, we can use 35 years of pooled cross-sections. The variables in Table 1 are used for the APC analyses.

Table 1: Variables of the data set

| Variable | Label | Description |
| --- | --- | --- |
| Country number | coun | A numeric code for every country |
| Country name | country |  |
| ISO country code | iso | ISO 3 character code |
| Sex | sex | Male indicator |
| 5 Year intervals | y5 | 5 year intervals of the years 1970 to 2005 |
| 5 year birth cohorts | g5 | 5 year intervals of cohorts born between 1895 to 1985 |
| 5 Year age intervals | a5 | 5 year age intervals 20 to 75 |
| 5 Year birth cohorts | coh | 5 year intervals of cohorts born between 1895 to 1985 |
| Suicide rate | r | Logged suicide rate |
| Number of suicide deaths | dsuic | suicide death count |
| Population size | pop | Population at risk |
| Number of deaths | deaths | Death count |
| Country cohort years | coun_coh | 5 years country and cohort intervals |
